# Chromosome-Level Genome Assembly of the Cape Cliff Lizard (*Hemicordylus capensis*)

**DOI:** 10.1093/gbe/evad001

**Published:** 2023-01-10

**Authors:** Henrique G Leitão, Genevieve Diedericks, Chris Broeckhoven, Simon Baeckens, Hannes Svardal

**Affiliations:** Department of Biology, University of Antwerp, 2610 Antwerp, Belgium; Department of Biology, University of Antwerp, 2610 Antwerp, Belgium; Department of Botany and Zoology, Stellenbosch University, Matieland Stellenbosch, South Africa; Department of Biology, University of Antwerp, 2610 Antwerp, Belgium; Department of Biology, University of Antwerp, 2610 Antwerp, Belgium; Department of Biology, Ghent University, 9000 Ghent, Belgium; Department of Biology, University of Antwerp, 2610 Antwerp, Belgium; Naturalis Biodiversity Center, Leiden, The Netherlands

**Keywords:** Cordylidae, HiFi, reference genome, Scincomorpha, sex determination, squamates

## Abstract

Squamates represent a highly diverse and species-rich vertebrate group that is remarkably understudied from a genomic perspective. A scarcity of genomic data is particularly evident for scincomorph lizards, which encompass over 10% of all living squamates, and for which high-quality genomic resources are currently lacking. To address this knowledge gap, we present the first chromosome-level reference genome for this group, generated from a male Cape cliff lizard (*Hemicordylus capensis*), using highly accurate PacBio HiFi long-read sequencing data, long-range Omni-C chromosomal conformation capture data and transcriptomic data for annotation. The rHemCap1.1 genome assembly spans 2.29 Gb, with a scaffold N50 of 359.65 Mb, and includes 25,300 protein-coding genes, with a BUSCO completeness score of 95.5% (sauropsida_odb10). We have generated the most contiguous and complete chromosome-level squamate reference genome assembly publicly available to date. Furthermore, we used short-read resequencing of 35 males and females and applied a differential coverage approach to infer the sex-determination system of the species, which was previously unknown. Our results suggest this species has XX/XY sex chromosomes, representing the first evidence of sex determination in the family Cordylidae. This reference genome will help to establish this species as an evolutionary model for studying variation in body armor, a key trait in cordylids and other squamate groups. Lastly, this is the first squamate reference genome from a continental African species and, as such, represents a valuable resource not only for further evolutionary research in cordylids but also in closely related groups.

**Significance**

We generated a high-quality, chromosome-level, annotated reference genome for the Cape cliff lizard, *Hemicordylus capensis*. This genome represents the most complete and contiguous chromosome-scale squamate assembly to date and the first genome assembly for the infraorder Scincomorpha. We also present the first evidence of the sex-determination system in cordylids, suggesting a XX/XY system for this species. This assembly represents the first reference genome for a continental African squamate and is an essential resource for evolutionary research in closely related species and families.

## Introduction

Squamates are the most diverse and species-rich group of reptiles (Vitt and Caldwell 2014; Simões and Pyron 2021) and the second-largest extant vertebrate order. They show variation in numerous ecologically relevant traits such as limblessness (Leal and Cohn 2018), coloration (Stuart-Fox et al. 2021), and chromosome number (Waters et al. 2021). Despite this group's exceptional phenotypic diversity, evolutionary research on its members is limited by a lack of high-quality genomic resources. To date, there are only 72 squamate reference genomes publicly available on NCBI (Sayers et al. 2021, accessed December 20, 2022), which is an order of magnitude lower when compared with similar-sized groups such as the order Aves (734 reference genomes) or the clade Percomorphaceae (711 reference genomes; Sayers et al. 2021, accessed December 20, 2022). African squamates are especially poorly represented, with only the Madagascar ground gecko (Hara et al. 2018) present on NCBI (Sayers et al. 2021), and there is currently no genome assembly available for a continental African squamate species.

Within squamates, the infraorder Scincomorpha includes the families Cordylidae, Gerrhosauridae, Scincidae, and Xantusiidae, forming a sister group to all other squamates excluding dibamids and gekkotans and encompassing over 10% of all living squamates (Pyron et al. 2013; Vitt and Caldwell 2014). The family Cordylidae contains 68 species (Uetz et al. 2022) widely distributed across sub-Saharan Africa, from South Africa to Ethiopia (Stanley et al. 2011; Nielsen and Colston 2014). Cordylids are ecologically and morphologically diverse and are composed of the subfamilies Platysaurinae and Cordylinae (Stanley et al. 2011). The latter have evolved viviparity and a distinct appearance, characterized by the expression of osteoderms which constitute protective body armor (Broeckhoven et al. 2016, 2018). Cordyline species differ greatly in this trait, and a reduction in body armor is thought to have evolved multiple times independently within the group (Stanley et al. 2011).

Given the lack of molecular data available for cordylids, the sex-determination system of this group remains unknown. Recently, the first evidence on the sex-determination systems of Gerrhosauridae (the sister group to Cordylidae) and Xantusiidae was published, suggesting a ZZ/ZW system shared among these groups (Kostmann et al. 2020; Kratochvíl et al. 2020; Nielsen et al. 2020). However, concrete evidence on the sex-determination system in cordylids is still lacking.

Among cordylids, the Cape cliff lizard, *Hemicordylus capensis*, exhibits remarkable variation in body armor, including between sexes and among populations (Stanley et al. 2011; Broeckhoven et al. 2017). It is an endemic species to South Africa, with a distribution that spans the Cape Fold Mountains in the Western Cape region, where it occupies highly diverse environments (Stanley et al. 2011). As such, this species provides an ideal model to study the rapid evolution of variation in body armor, a trait that fundamentally impacts many aspects of ecological performance (Broeckhoven et al. 2018).

We integrated PacBio HiFi long-read sequencing, Omni-C long-range proximity ligation, and RNA-sequencing data to generate a high-quality chromosome-level annotated genome assembly of the Cape cliff lizard, *H. capensis*. Furthermore, we used Illumina short-read population sequencing data to shed light on the sex-determination system in cordylids. This reference genome is a valuable resource that will enable evolutionary genomics studies for closely related species and families.

## Results and Discussion

### Genome Assembly

PacBio long-read sequencing yielded over 12.5 million HiFi reads across 6 SMRT Cells 8M, for a cumulative total of 98.14 Gb (supplementary table S1, Supplementary Material online). *K*-mer analysis (*k* = 32) showed a *k*-mer distribution profile consistent with the theoretical diploid model (bimodal distribution) and gave an estimated genome size of 2,276,570,909 bp (supplementary fig. S1, Supplementary Material online).

**Fig. 1.**
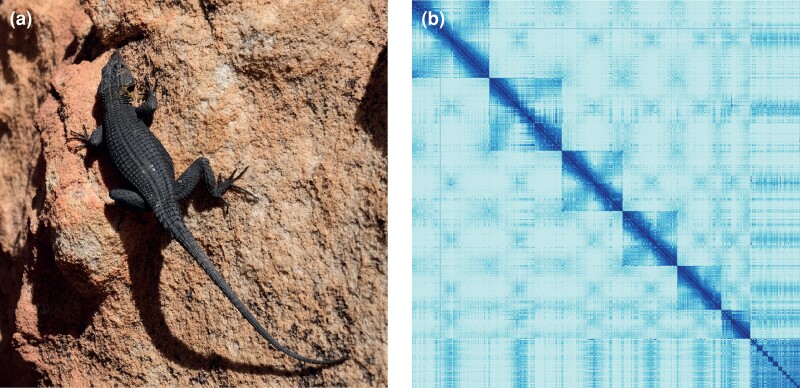
(*a*) Representative image of *Hemicordylus capensis*. (*b*) Hi-C contact map of the rHemCap1.1 assembly visualized in PretextMap. Chromosomes are ordered by size, from top left to bottom right.

We generated a genome assembly from a male *H. capensis* (fig. 1*a*) by integrating paired-end chromosomal conformation capture data (Omni-C, supplementary table S2, Supplementary Material online) with highly accurate long-read sequencing (HiFi, supplementary table S1, Supplementary Material online). After haplotypic duplication removal, scaffolding, and manual curation, the resulting reference genome had a total length of 2.29 Gb, with a contig N50 of 160.69 Mb and a scaffold N50 of 359.65 Mb (supplementary table S3, Supplementary Material online), exceeding the standards for high-quality genome assemblies stated by the Vertebrate Genomes Project (Rhie et al. 2021). Most scaffolds (99.94%) were assigned to 17 chromosomes, numbered in descending order of sequence length (fig. 1*b*). As in other reptiles (Waters et al. 2021), the *H. capensis* genome comprises both macro- (6) and microchromosomes (11), with a diploid number identical to that of other cordylids and gerrhosaurids for which there is karyologic evidence (Olmo and Odierna 1980). The final assembly had a QV score of 61.49 (probability of <1 incorrect base call per 1 Mb) and a BUSCO completeness score of 95.5% using the sauropsida_odb10 gene set. This is the most contiguous and complete publicly available chromosome-scale squamate reference genome to date (supplementary fig. S2 and table S4, Supplementary Material online).

### Genome Annotation

Repeat annotation detected 55.9% of the *H. capensis* genome as repetitive, with long interspersed nuclear elements identified as the most abundant repetitive element class (19.11%), followed by long terminal repeat elements (4.93%), simple repeats (4.89%), DNA transposons (2.95%), and short interspersed nuclear elements (2.37%) (supplementary table S5, Supplementary Material online). Genome annotation predicted 25,300 genes and mRNAs, of which 15,999 (63.24%) had matches to a PFAM domain (supplementary table S6, Supplementary Material online). Evaluation of the resulting set of proteins with BUSCO showed a completeness score of 89.8% with the sauropsida_odb10 data set (supplementary table S6, Supplementary Material online).

### Mitochondrial Genome Assembly

The mitochondrial genome assembled with MitoHiFi had a final size of 17,116 bp. It consists of 23 transfer RNAs and 14 protein-coding genes and has a base composition of A = 32.68%, C = 27.24%, G = 13.5%, T = 26.58%.

### Synteny Analysis

We conducted a pairwise synteny analysis between *H. capensis*, *Phrynosoma platyrhinos*, and *Sphaerodactylus townsendi* to investigate how conserved chromosome structure is between these distantly related species. Synteny is largely conserved along the macrochromosomes across the three species (supplementary fig. S3, Supplementary Material online), especially between *H. capensis* and *P. platyrhinos*. Notably, chromosomes 12 and 16 are homologous to chromosome 16 in *S. townsendi*, but corresponding syntenic regions were not identified in *P. platyrhinos.* Chromosome 3 is syntenic to chromosome 3 in *P. platyrhinos*, which in turn is homologous to the long arm of chromosome 1, and chromosomes 6 and 9 in chicken (Koochekian et al. 2022). Our results suggest that this fusion may be more ancient than previously suggested and ancestral in Unidentata rather than in Toxicofera (Deakin et al. 2016).

### Identification of the sex-determination System of *H. capensis*

**Fig. 2.**
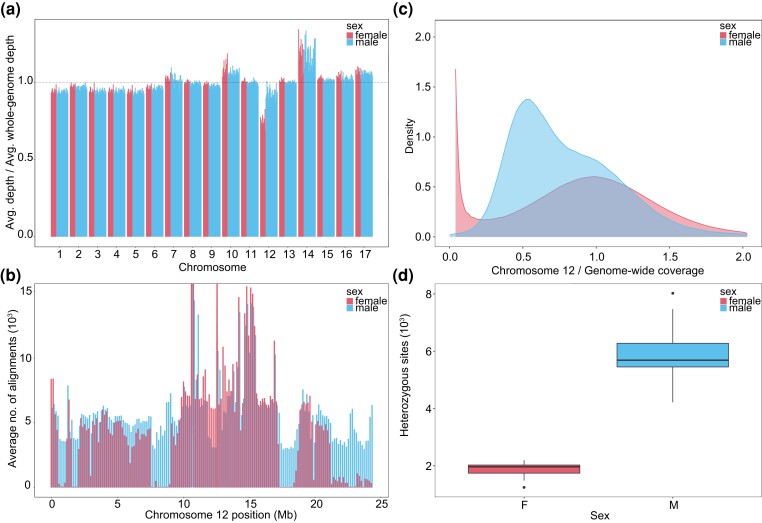
(*a*) Ratio between the average depth of coverage on a given chromosome and the average whole-genome depth for males and females. Each bar represents an individual. (*b*) Average number of alignments for each sex in 150 kb windows along the 12th chromosome. Due to regions with extreme average alignment values, the *y*-axis upper limit was set to 15,000 alignments for representation purposes. (*c*) Density plot of average chromosome 12 per-base coverage normalized by genome-wide average coverage for males and females. Due to an extreme frequency of positions lacking coverage in females and the presence of some regions with extremely high coverage in both sexes, the *x*- and *y*-axis upper limits were set to 2 for representation purposes. (*d*) Number of heterozygous sites in males and females in regions of the 12th chromosome that were present in both sexes.

To identify the sex chromosome and infer the sex-determination system in *H. capensis*, we used a differential coverage approach, comparing whole-genome sequencing data from males and females. We short-read sequenced 35 individuals with an average genome-wide coverage of 4.4X (range 1.3—11.8X; supplementary table S7, Supplementary Material online). The 12th largest chromosome showed a marked difference in coverage between males and females (Welch's *t* = −13.7, *P* < 10^−10^; fig. 2*a*). This difference resulted from the female sequences not aligning to specific regions on that chromosome (fig. 2*b*). In other regions of chromosome 12, females had similar, but generally slightly lower, coverage compared with males (fig. 2*b*). Of the eight juveniles for which sex was unknown, four were classified as female due to lower coverage on chromosome 12 and a mapping signal identical to that of the females (supplementary table S7, Supplementary Material online). The coverage distribution on chromosome 12 (normalized by the genome-wide average coverage) showed a bimodal distribution in females, with peaks close to 0 and 1× the genome-wide average in females, whereas males had a higher peak close to 0.5× and a lower peak close to 1× the genome-wide average (fig. 2*c*). In the regions present in both males and females, heterozygosity was significantly higher in males (Welch's *t* = −19.4, *P* < 10^−18^; fig. 2*d*). In the regions present only in males, average male coverage was approximately half (55%) of that on the rest of chromosome 12 (Welch's *t* = −427, *P* ≈ 0). Together, these results suggest that chromosome 12 corresponds to a Y chromosome with extensive pseudo-autosomal regions. Therefore, we conclude that *H. capensis* has an XX/XY sex-determination system, with males being the heterogametic sex. This result is particularly interesting given the recent identification of ZZ/ZW systems in the two other Cordylomorpha families, Gerrhosauridae and Xantusiidae (Kostmann et al. 2020; Kratochvíl et al. 2020; Nielsen et al. 2020). As in other squamate clades (Augstenová et al. 2018; Nielsen et al. 2018; Keating et al. 2021), sex-determination systems appear to be exceptionally dynamic in this group, and further studies of species-specific scincomorph sex-determination systems are warranted.

## Materials and Methods

### Sample Collection, DNA Extraction, and Sequencing

The reference genome was sequenced and assembled from a wild-caught male Cape cliff lizard (rHemCap1) collected at Gifberg, Western Cape, South Africa. High-molecular weight DNA was extracted from brain, liver, and gonadal tissue using the Nanobind Tissue Big DNA kit (Circulomics) and sequenced on six PacBio Sequel IIe SMRT Cells 8M (HiFi) by Inqaba Biotechnical Industries, Pretoria, South Africa.

### Omni-C Library Preparation and Sequencing

Two Omni-C libraries were prepared from blood collected from the reference male using the Dovetail Omni-C kit according to the manufacturer's protocol. The fragment size distribution and library concentration were assessed by VIB-UAntwerpen using a Fragment Analyzer (Agilent Technologies) and a Qubit Fluorometer (Thermo Fisher Scientific, Waltham, MA, USA), respectively. Paired-end 150 bp sequencing was performed by GENEWIZ on an Illumina NovaSeq platform.

### RNA-Sequencing

Total RNA was extracted from five tissues (brain, gonads, heart, liver, and muscle) for the reference male and a female from the same population, using the TRIzol™ Reagent and Phasemaker™ Tubes Complete System (Invitrogen) and following the manufacturer's instructions. RNA integrity was checked using a Fragment Analyzer. Library preparation with poly(A) enrichment and paired-end 150 bp sequencing was performed by GENEWIZ on an Illumina NovaSeq platform.

### Genome Assembly

Firstly, PacBio HiFi reads were filtered to remove remnant adapter sequences with HiFiAdapterFilt v2 (Sim et al. 2022), and the expected genome size was calculated via genome profiling with the filtered HiFi data, using a *k*-mer spectra analysis (*k* = 32) with meryl v1.3 (https://github.com/marbl/meryl) and GenomeScope 2.0 (Ranallo-Benavidez et al. 2020). The genome was assembled with hifiasm v0.16.1-r375 (Cheng et al. 2022) with Omni-C data integration and setting the primary option. This resulted in primary and alternate assemblies; the former was used for all remaining analyses. Haplotypic duplication was removed from the primary assembly with purge_dups v1.2.5 (Guan et al. 2020). Using the Omni-C data, the primary assembly was scaffolded with yahs v1.2a (Zhou et al. 2023). Briefly, the Omni-C reads were aligned to the assembly with bwa mem v0.7.17-r1188 (Li 2013). From the mapped reads, valid ligation events were recorded, and PCR duplicates were removed using pairtools v0.3.0 (https://github.com/open2c/pairtools), producing the .bam file that was used for scaffolding. Contamination was checked with BlobToolKit (Challis et al. 2020) and the NCBI Foreign Contamination Screen (https://github.com/ncbi/fcs). Manual curation was done using PretextMap v0.1.9 (https://github.com/wtsi-hpag/PretextMap), PretextView v0.2.5 (https://github.com/wtsi-hpag/PretextView), and PretextSnapshot v0.0.4 (https://github.com/wtsi-hpag/PretextSnapshot). Gfastats v1.3.1 (Formenti et al. 2022) was used to obtain assembly contiguity metrics, and merqury v1.3 (Rhie et al. 2020) was used to assess base-level accuracy. We used BUSCO v5.2.2 (Manni et al. 2021) with the default BUSCO_MetaEuk workflow option and the sauropsida_odb10 data set to evaluate assembly completeness and to compare this with all available squamate reference genomes (supplementary table S4, Supplementary Material online). N50 and aUN (https://lh3.github.io/2020/04/08/a-new-metric-on-assembly-contiguity) values were calculated for each squamate assembly. The mitochondrial genome was assembled using MitoHiFi v2.2 (Uliano-Silva et al. 2021), with the mitochondrial genome of *Smaug warreni* (NC_005962) as the most closely related reference available.

### Genome Annotation

To identify and mask repetitive sequences, we used RepeatModeler2 v2.0.3 (Flynn et al. 2020) for de novo identification of transposable elements. These regions were then masked along with known metazoan repeats in the assembly using RepeatMasker v4.1.2 (Smit et al. 2013), and a repeat element landscape plot was generated (supplementary fig. S4, Supplementary Material online). Following repeat annotation, we conducted gene annotation. Firstly, RNA-seq reads were filtered with fastp v0.23.2 (Chen et al. 2018) in default mode and then mapped to the genome with STAR v2.7.10a (Dobin et al. 2013). Then, gene models in the masked assembly were predicted with BRAKER2 v2.1.6 using GeneMark-EX, and AUGUSTUS v.3.4.0 (Brůna et al. 2021), with the RNA-seq mapped reads as evidence and the pre-trained parameter set for chicken, the closest available reference taxon. TSEBRA v1.0.3 (Gabriel et al. 2021) was used with default parameters to select the best predicted gene models based on RNA-seq evidence. Next, we ran InterProScan v5.57-90.0 (Jones et al. 2014) and eggNOG mapper v2.1.9 (Cantalapiedra et al. 2021) for functional annotation. All annotation information was integrated with funannotate v.1.8.13 (https://github.com/nextgenusfs/funannotate) to produce a final annotation file. Lastly, we evaluated the annotation completeness by running BUSCO v5.2.2 in protein mode with the sauropsida_odb10 data set.

### Synteny Analysis

To investigate synteny between *H. capensis* and other squamates, we used GENESPACE v0.9.3 (Lovell et al. 2022) with OrthoFinder v2.5.4 (Emms and Kelly 2019) in “default” mode and the genome annotations and protein sequences of *S. townsendi* (Pinto et al. 2022) and *P. platyrhinos* (Koochekian et al. 2022).

### Sex-Determination Analysis

For Illumina short-read sequencing, DNA of 35 additional individuals (20 males, 7 females, and 8 juveniles for which sex was unknown) from the same population as the reference male was extracted from tail tissue with the DNeasy Blood & Tissue Kit (QIAGEN) following the manufacturer's instructions. Library preparation and paired-end 150 bp sequencing was performed by GENEWIZ on an Illumina NovaSeq platform. Raw reads were filtered with fastp v0.23.2 (Chen et al. 2018) and aligned to the reference genome with BWA MEM v0.7.17-r1188. Coverage was calculated for each sample with SAMtools v1.14 (Danecek et al. 2021), and BEDTools v2.30.0 (Quinlan and Hall 2010) was used to count alignments per sample along 150 kb windows along the 12th chromosome with the MultiCoV function. To investigate heterozygosity differences between sexes, variants on the 12th chromosome were called with BCFtools v1.14 (Danecek et al. 2021) with minimum mapping quality 30 and minimum base quality 30. Variants were filtered with VCFtools v0.1.16 (Danecek et al. 2011) to include only sites present in at least 95% of individuals, with a minimum depth of one read and a maximum depth of 50 reads. Heterozygosity was then calculated for each individual with plink v2.00a2.3LM (Chang et al. 2015) and tested for a difference between sexes with Welch's *t*-test.

## Supplementary Material

evad001_Supplementary_Data

## Data Availability

The primary and alternate genome assemblies, and all raw sequencing data, have been deposited in the NCBI databases under BioProject accessions PRJNA899811, PRJNA899810, and PRJNA902320. The genome annotation is available on the Zenodo data repository under accession doi:10.5281/zenodo.7330055.
