# Supplementary material for: Chromosome-Level Genome Assembly of the Cape Cliff Lizard (Hemicordylus capensis)
Source: Genome Biol Evol. 2023 Jan 10;15(2):evad001. doi: 10.1093/gbe/evad001 (PMC9907493; doi:10.1093/gbe/evad001)
Supplement: evad001_Supplementary_Data [file evad001_supplementary_data.zip › Leitao_2022_Supp_Figs.docx]

**Supplementary Material**

**Chromosome-level genome assembly of the Cape cliff lizard (*Hemicordylus capensis*)**

Henrique G. Leitão^1*^, Genevieve Diedericks^1,2^, Chris Broeckhoven^1^, Simon Baeckens^1,3^, Hannes Svardal^1,4*^

**Affiliations:**

^1^Department of Biology, University of Antwerp, 2610 Antwerp, Belgium

^2^Department of Botany & Zoology, Stellenbosch University, Private Bag X1, Matieland 7602, Stellenbosch, South Africa

^3^Department of Biology, Ghent University, 9000 Ghent, Belgium

^4^Naturalis Biodiversity Center, 2333 CR Leiden, Netherlands

**Supplementary Figures**


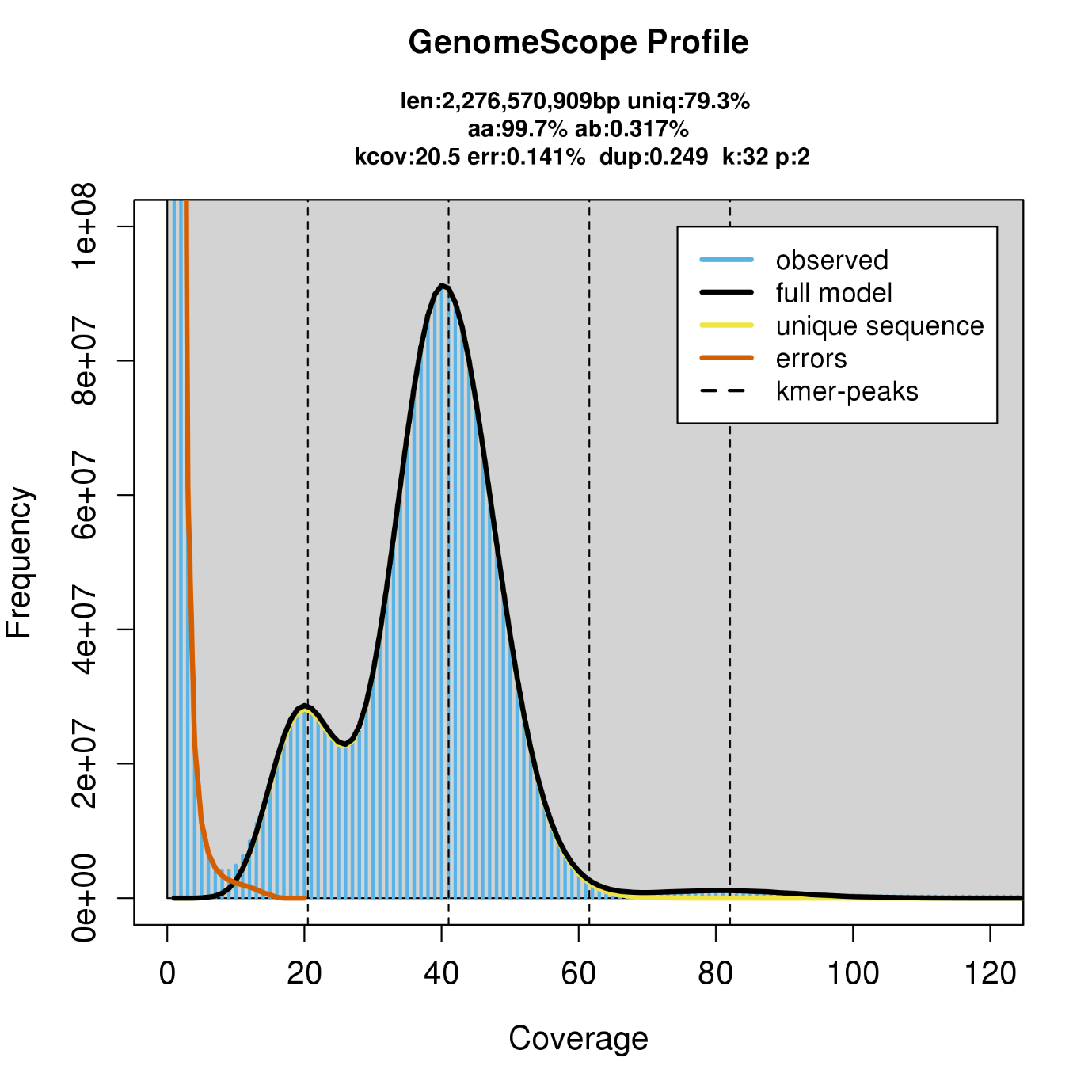
**Fig. S1— GenomeScope2.0 32-mer profile.** K-mer count distribution (k = 32) calculated with GenomeScope2.0, including model results. The first peak at approximately 20-fold coverage represents the heterozygous coverage, while the second peak at approximately 40-fold coverage represents the homozygous coverage. Estimated heterozygosity is 0.317 %.

**
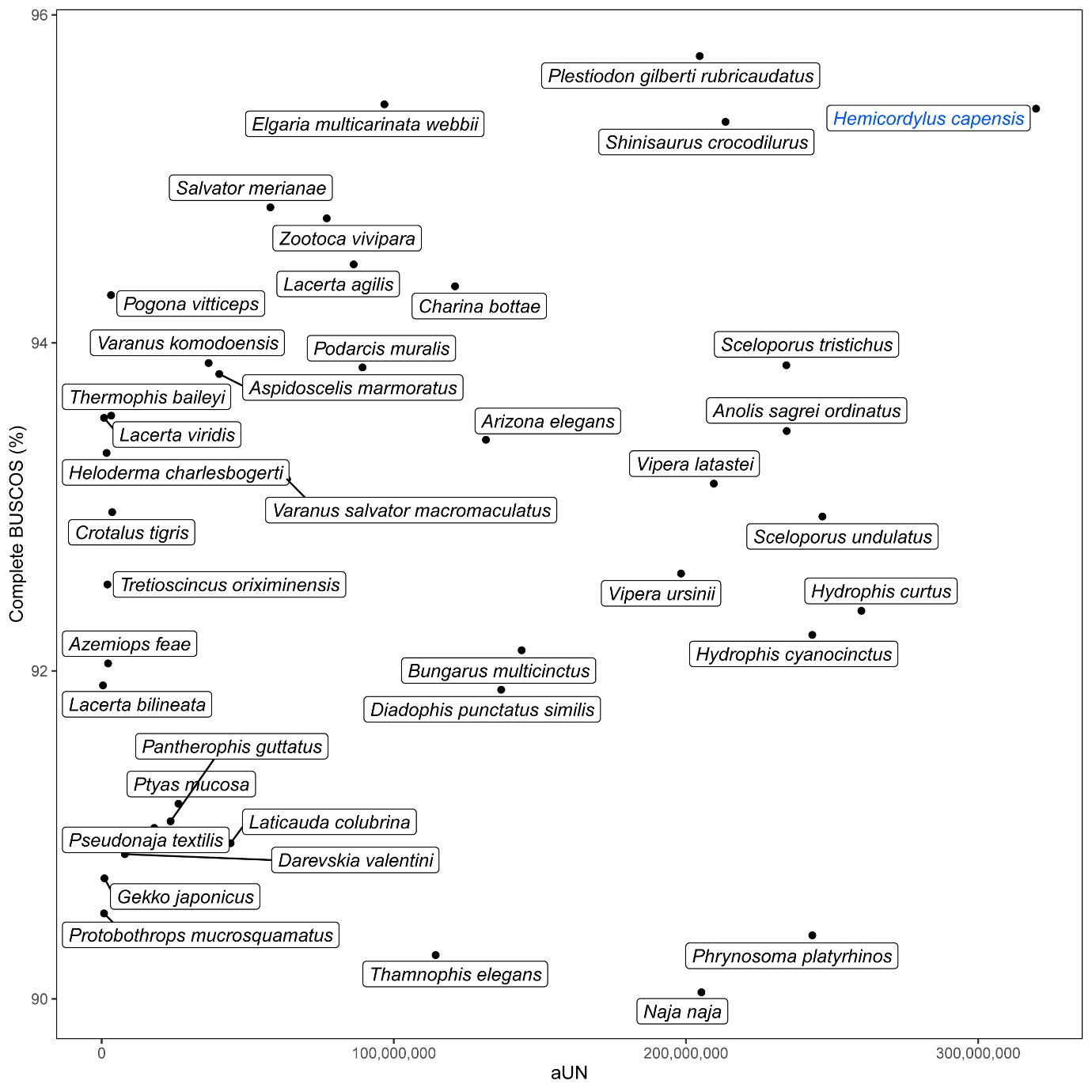
Fig. S2— Contiguity and completeness of squamate genome assemblies.** Percentage of complete BUSCOs (sauropsida_odb10) and aUN values for squamate reference genomes with ≥ 90% complete BUSCOs.


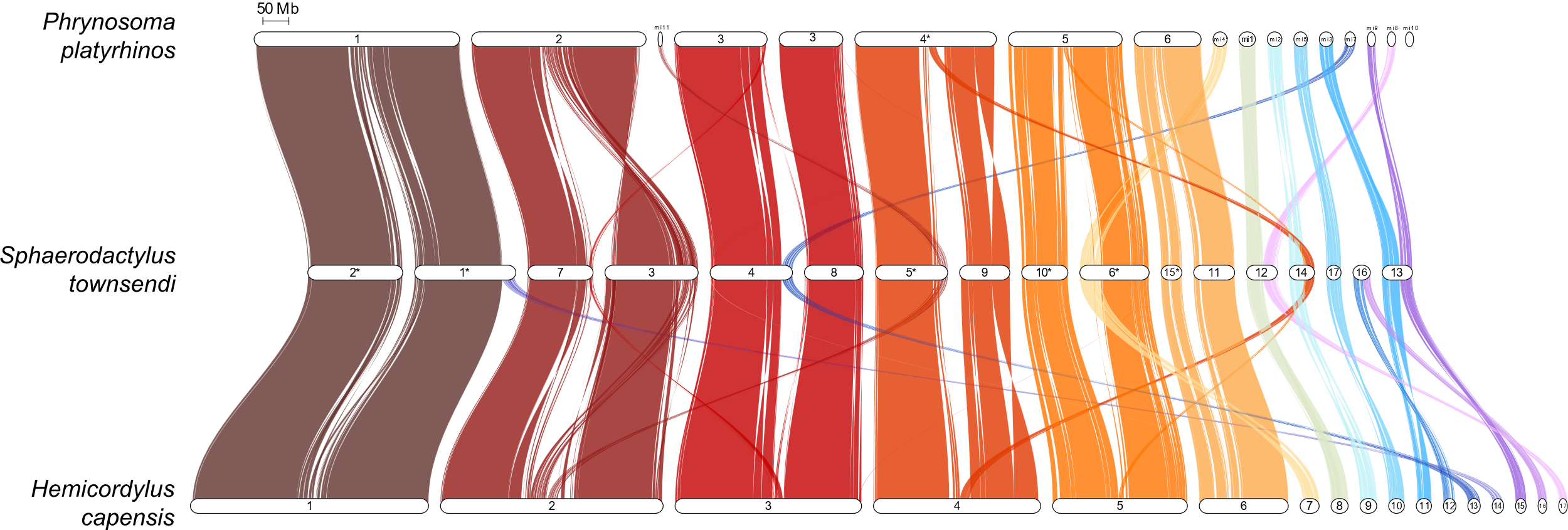


**Fig. S3— Synteny analysis.** Chromosomal structure comparison between *Hemicordylus capensis*, *Sphaerodactylus townsendi*, and *Phrynosoma platyrhinos*. Chromosomes marked with an asterisk have been inverted to match the orientation of homologous chromosomes.

**
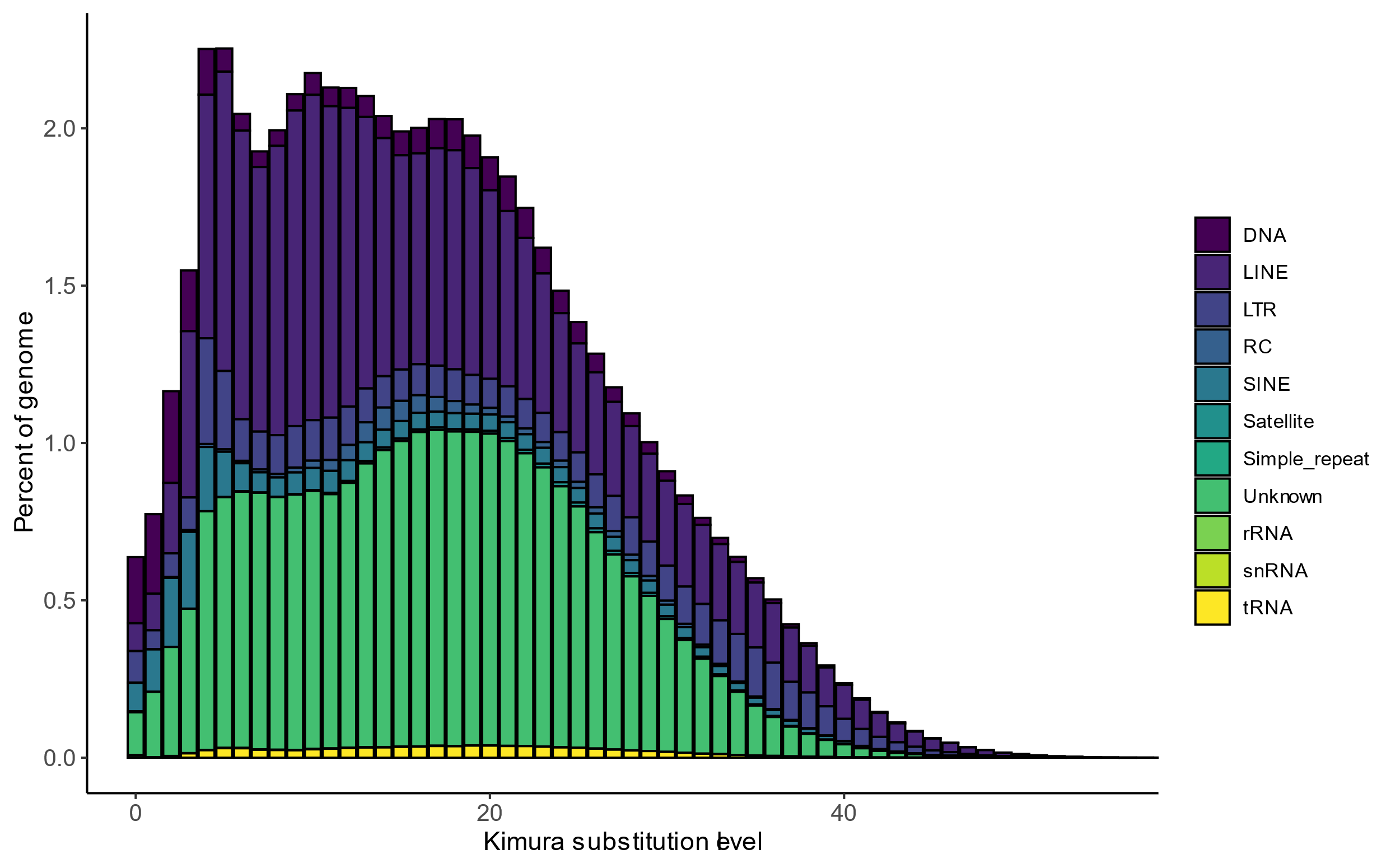
**

**Fig. S4— rHemCap1.1 repeat landscape.** Percentage of genome and sequence divergence (Kimura substitution level) are shown for each repetitive element class.
